# Diagnostic and cost‐effectiveness of axial skeleton MRI in staging high‐risk prostate cancer

**DOI:** 10.1002/bco2.210

**Published:** 2023-01-31

**Authors:** Omar El‐Taji, Hannah Evans, Vandan Arora, Suzanne Amin, Manal Kumar, Thiagarajan Nambi Rajan

**Affiliations:** ^1^ Department of Urology Wirral University Teaching Hospitals Wirral UK; ^2^ Department of Radiology Wirral University Teaching Hospitals Wirral UK; ^3^ School of Medicine University of Bolton Bolton United Kingdom

**Keywords:** bone metastasis, cost analysis, high‐risk, magnetic resonance imaging, prostate cancer

## Abstract

**Introduction:**

Current literature suggests that axial skeleton magnetic resonance imaging (AS‐MRI) is more sensitive than Tc 99m bone scintigraphy (BS) for detecting bone metastases (BM) in high‐risk prostate cancer (PCa). However, BS is still widely performed. Its diagnostic accuracy has been studied; however, its feasibility and cost implications are yet to be examined.

**Methods:**

We reviewed all patients with high risk PCa undergoing AS‐MRI over a 5‐year period. AS‐MRI was performed on patients with histologically confirmed PCa and either PSA > 20 ng/ml, Gleason ≥8, or TNM Stage ≥T3 or N1 disease. All AS‐MRI studies were obtained using a 1.5‐T AchievaPhilips™MRI scanner. We compared the AS‐MRI positivity and equivocal rate with that of BS. Data were analysed according to Gleason score, T‐stage and PSA. Multivariate logistic regression analyses were used to quantify the strength of association between positive scans and clinical variables. Feasibility and burden of expenditure was also evaluated.

**Results:**

Five hundred three patients with a median age of 72 and a mean PSA of 34.8 ng/ml were analysed. Eighty‐eight patients (17.5%) were positive for BM on AS‐MRI (mean PSA 99 [95% CI 69.1–129.9]). Comparatively 409 patients (81.3%) were negative for BM on AS‐MRI (mean PSA 24.7 (95% CI [21.7–27.7]) (*p* = 0.007); 1.2% (*n* = 6) of patients had equivocal results (mean PSA 33.4 [95% CI 10.5–56.3]). There was no significant difference in age (*p* = 0.122) between this group and patients with a positive scan, but there was a significant difference in PSA (*p* = 0.028), T stage (*p* = 0.006) and Gleason score (*p* = 0.023). In comparison with BS, AS‐MRI detection rate was equivalent or higher compared with the literature. Based on NHS tariff calculations, there would be a minimum cost saving of £8406.89. All patients underwent AS‐MRI within 14 days.

**Conclusion:**

The use of AS‐MRI to stage BM in high‐risk PCa is both feasible and results in a reduced burden of expenditure.

## 1 INTRODUCTION

Approximately 5%–10% of men with newly diagnosed prostate cancer (PCa) have bone metastases (BM).^1^ Bone represents the initial metastatic site in more than 80% of PCa patients; early detection is therefore imperative. Although greater awareness and prostate‐specific antigen (PSA) testing has shifted the burden of disease towards lower stage and tumour grade, a considerable number of men still present with metastatic prostate cancer. As age at diagnosis falls and life expectancy rises, we are now treating more advanced and higher grade cancers with curative intent.

Unfortunately, a proportion of patients who undergo radical treatment whether through hormones and radiotherapy or through radical prostatectomy will eventually die from metastatic disease.^2^ It is possible that better bone imaging could correctly identify patients with low‐volume metastatic disease at diagnosis in order to offer the most appropriate treatment options for these patients and redirect them to systemic therapy. NICE, EAU and AUA recommend bone imaging in patients with high‐risk disease.^3^, ^4^, ^5^, ^6^ Given the national prostate cancer audit (NPCA) revealed that 42% of newly diagnosed patients in England with PCa have high‐risk disease, finding an accurate yet cost‐effective imaging modality is essential.^7^

We still rely heavily on Tc 99m bone scintigraphy (BS) as the imaging modality of choice for detecting BM. Indeed, it has stood the test of time probably due to its accessibility, easy interpretation and relative cost. Studies however have shown its inferiority in the context of sensitivity in comparison with new imaging modalities.^8^, ^9^ BS sensitivity is low, because BS detects bone deposits from osteoblasts, not cancer cells.^10^ Areas of increased uptake are often classed as “equivocal,” “possible” or “likely”; these are a group of definitions that encompasses all cases in which imaging findings could not be classified confidently as metastatic or benign, regardless of the level of doubt. These areas often require targeted X‐rays (TXR) to distinguish benign from metastatic lesions. Despite this, it is not uncommon for patient to need further evaluation through CT or MRI.

Diffusion weighted whole body (WB‐MRI) and axial skeleton (AS‐MRI) magnetic resonance imaging have emerged as frontrunners with studies reporting increased sensitivity and specificity while avoiding radiation in comparison with BS for detecting BM in PCa.^11^, ^12^ In addition, MRI allows a quantitative evaluation of BM, which is currently deemed to be non‐measurable with BS ± TXR, owing to their poor spatial resolution.^13^ For over 20 years, its superiority over BS has been demonstrated; however, its limited availability and perceived cost has meant it has taken a back seat.

Our study aims to assess the diagnostic effectiveness, feasibility and cost implication of implementing a service which utilises AS‐MRI for the detection of BM in patients with high‐risk PCa. We compare our data to that available in the literature to demonstrate comparison.

## 2 METHODS

We reviewed all patients with PCa undergoing AS‐MRI for high‐risk disease over a 5‐year period. AS‐MRI was performed on patients with a confirmed diagnosis of PCa and either Gleason ≥8, PSA > 20 ng/ml or TNM radiological stage ≥T3 or N1 disease.^4^ All patients were treatment naïve. Patients were only included if they had a PSA and histologically confirmed adenocarcinoma of the prostate within 30 days of an AS‐MRI. Patients were excluded for data analysis if they did not have histologically proven prostate adenocarcinoma.

Patient demographics extracted included age, presenting PSA, radiological T stage and Gleason Grade. We used the highest PSA recording within 30 days of the AS‐MRI, and radiological T stage was based on multiparametric MRI prostate (mpMRI prostate). mpMRI prostate included axial, coronal and sagittal T2‐weighted images, axial T1‐weighted images, axial DWI and dynamic contrast enhancement, covering the prostate gland, seminal vesicles and the pelvis up to the aortic bifurcation. Systematic and/or targeted prostate biopsies were processed in accordance with the ISUP 2014 modified grading system.^12^

All patients underwent AS‐MRI with the exact same protocol. All AS‐MRI studies were obtained using a 1.5‐T Philips™Achieva/Ingenia MRI scanner with a Quadrature body coil, and included coronal T1 weighted, coronal Short T1 Inversion Recovery (STIR), axial diffusion weighted sequence images and sagittal modified Dixon sequences. Patients were scanned from vortex to knee. No intravenous contrast was given. A uro‐radiologist categorised scan findings as negative, positive or equivocal for BM. Patients were discussed at the multi‐disciplinary team meeting following their scan and a management plan was individualised according to clinical profile and performance status.

Time interval between requesting and performing the scan was calculated as well as time required for each AS‐MRI. As a comparison BS positivity rate was derived from studies of patients with high‐risk prostate cancer. For the estimation of upstream costs of AS‐MRI and BS, the UK national tariff system for 2020–2021 was used.^13^

Chi‐squared and Student's *t* tests were used to compare categorical and continuous variables respectively. Risk factors for BM was determined using a univariate analysis. Those variables that reached statistical significance (*p* < 0.05) were considered for the model. A multivariate stepwise logistic regression analysis was then performed to identify independent predictors of a positive AS‐MRI. For all statistical analyses, *p* < 0.05 was considered statistically significant.

## 3 RESULTS

A total of 549 patients underwent AS‐MRI at our centre over a 5‐year period. Forty‐six patients were excluded due to lack of histological confirmation of the diagnosis (*n* = 37) or due to lack of PSA within 30 days of AS‐MRI (*n* = 9). A total of 503 patients therefore fulfilled our criteria with a median age of 72 and a mean PSA of 34.8 ng/ml (95% CI 29.6–40.0) (Table 1).

**TABLE 1:** Clinical characteristics

Variables	All patients	Patients without BM	Patients with BM	*p* value	Patients with an equivocal AS‐MRI	*p* value^a^
Patient no. (%)	503	409 (81.3%)	88 (17.5%)	‐	6 (1.2%)	‐
Age, year				**0.031**		0.122
Median	72	70	72		73	
Range	45–87	45–86	52–87		71–83	
Clinical T stage, no. (%)				**<0.001**		**0.006**
Organ confined (<T3)	246 (48.9)	236 (57.7)	7 (7.6)		3 (50)	
Locally advanced (≥T3)	257 (51.1)	173 (42.3)	81 (92)		3 (50)	
PSA ng/ml				**<0.001**		**0.028**
Mean (95% CI)	34.8 (29.6–40.0)	24.7 (21.7–27.7)	99 (69.1–129.9)		33.4 (10.5–56.3)	
PSA, ng/ml, no. (%)				‐		‐
<10	146 (29.1)	138 (33.7)	8 (9.1)		0	
10.1–19.9	147 (29.2)	135 (33)	9 (10.2)		3 (50)	
20–49.9	120 (23.9)	102 (24.9)	16 (18.1)		2 (33.3)	
>50	85 (16.9)	30 (7.3)	54 (61.4)		1 (16.7)	
Gleason score, no. (%)				**<0.001**		**0.023**
≤6	26 (5.2)	26 (6.4)	0		0	
3 + 4	117 (23.3)	111 (27.1)	6 (6.8)		0	
4 + 3	35 (6.9)	23 (5.6)	9 (10.2)		3 (50)	
≥8	325 (64.8)	249 (60.1)	73 (82.9)		3 (50)	

^a^ Compared with patients with BM.

**FIGURE 1:**
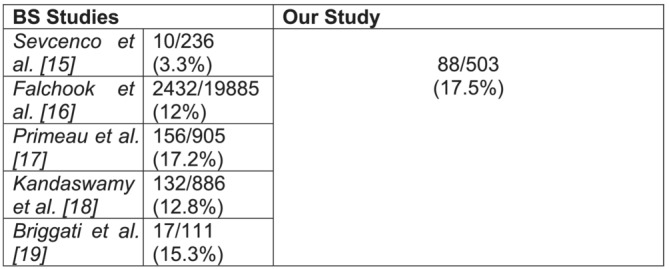
BS studies' rates of BM in patients with newly diagnosed high‐risk PCa

There were 88 patients (17.5%) who were positive for BM on AS‐MRI with a median age of 72 and a mean PSA of 99 ng/ml (95% CI 69.1–129.9). Comparatively 409 patients (81.3%) were negative for BM on AS‐MRI with a median age of 70 and mean PSA of 24.7 ng/ml (95% CI 21.7–27.7). Both age (*p* = 0.031) and PSA level (*p* < 0.001) were significantly higher in patients with a positive AS‐MRI on a two‐tailed *t* test. Moreover, patients with BM showed a significantly higher rate of locally advanced PCa (*p* < 0.001) as well as higher biopsy Gleason score distribution (*p* < 0.001) compared with patients without BM. In comparison with BS data for high‐risk disease, our BM detection rate was equivalent or higher compared with the literature (Figure 1).

Univariate logistic regression analyses were used to quantify the strength of association between positive scans and the available clinical variables (Table 2). Our analysis demonstrated that initial PSA level, radiological stage and Gleason score were all independent predictors of BM. Multivariate logistic regression analysis employing these independent predictors established that a PSA > 20 ng/ml (*p* = 0.042), locally advanced disease (*p* < 0.001) and Gleason score of ≥4 + 3 (*p* = 0.029) were independent predictors of BM (Table 2).

**TABLE 2:** Univariate and multivariate analysis of clinical variables

Clinical variable	OR (95% CI)	*p* value
Univariate analysis		
PSA		
PSA < 10	Reference	‐
PSA 10.1–19.9	1.80 (0.11–12.65)	0.046
PSA 20–49.9	2.95 (1.53–29.23)	0.002
PSA > 50	28.02 (7.16–98.31)	<0.001
T stage		
Organ confined	Reference	‐
Locally advanced	8.54 (2.31–14.24)	<0.001
Gleason score		
G < 7	Reference	
G 3 + 4	5.49 (2.74–68.43)	0.031
G 4 + 3	12.84 (5.18–94.71)	<0.001
G ≥ 8	25.72 (6.42–122.16)	<0.001
Multivariate analysis		
PSA		
PSA < 10	Reference	‐
PSA 10.1–19.9	1.1 (0.18–6.31)	0.62
PSA 20–49.9	2.08 (0.98–14.17)	**0.042**
PSA > 50	16.52 (5.56–78.31)	**<0.001**
T stage		
Organ confined	Reference	‐
Locally advanced	6.02 (4.21–139.51)	**<0.001**
Gleason score		
G < 7	Reference	‐
G 3 + 4	2.04 (0.32–16.47)	0.0511
G 4 + 3	4.7 (2.75–32.11)	**0.029**
G ≥ 8	11.32 (3.22–65.75)	**<0.001**

**FIGURE 2:**
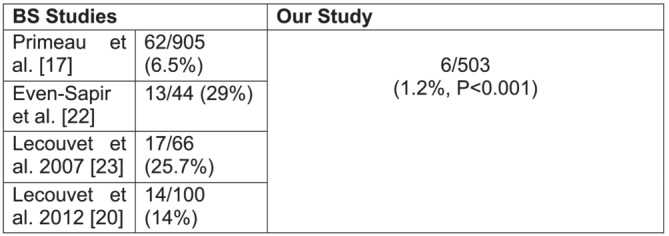
BS studies' rates of equivocal results in patients with newly diagnosed high‐risk PCa

Only 1.2% (*n* = 6) of patients had equivocal results; these patients had a mean PSA of 33.4 ng/ml (95% CI 10.5–56.3). One of these patients had myelofibrosis which meant interpretation of imaging was difficult and the remainder had a solitary nonspecific small lesion (<1 cm). These patients underwent CT, which confirmed no BM. There was no significant difference in age (*p* = 0.122) between this group and patients with a positive scan, but there was a significant difference in PSA (*p* = 0.028), Gleason score (*p* = 0.023) and T stage (*p* = 0.006) with those with a positive scan trending towards a lower PSA, Gleason grade and T stage. Our subanalysis showed that in comparison with previous studies, our rate of an equivocal AS‐MRI was significantly less than that for BS (*p* < 0.001) (Figure 2).

All scans were performed within 50 min. The average waiting time for a scan (from scan request to scan performed) was 12 days (95% CI 5–22), which was less than the national waiting target for cancers (14 days).

Based on NHS tariff calculations, the cost evaluation indicated each AS‐MRI (RD06Z) cost £173.86 resulting in a net spend for this patient cohort of £87 451.58. If we were to include the patients who had equivocal results on AS‐MRI and subsequently underwent CT (RD20A), this would result in an additional cost of £440.88 (£73.48 per CT). This equates to a total net expenditure of £87 892.46. In comparison, each BS (RN15A) costs £191.45, which would result in a minimum net expenditure of £96 299.35. This would result in a minimum cost saving of £8406.89.

## 4 DISCUSSION

Accurate diagnosis of BM is crucial in identifying patients with newly diagnosed high‐risk PCa in order to recommend the most appropriate treatments. BS identifies BM at an advanced stage of tumour infiltration when osteoblastic response to tumour deposition occurs.^9^ The interpretation of negative scans is therefore not completely reliable. In comparison, the advantage of MRI lies in its capability to identify cells seeded into the haematopoietic marrow and its adipose cells, consequently identifying BM earlier before osteoblastic response becomes visible on BS.^11^ The superiority of MRI over BS is well established for the detection of BM in PCa and other malignancies yet the use of BS for staging is still widely practised. It remains somewhat unclear why existing uro‐oncological guidelines have adapted data from BS studies with methodological problems, while seemingly superior modalities are yet to be recommended.

In PCa, MRI has been suggested as a cost‐effective substitute to conventional imaging in terms of sensitivity and specificity. The limited specificity of BS translates to the need for additional imaging by way of TXR, or even MRI or CT to characterise equivocal lesions.^7^, ^8^ Although this improves both sensitivity and specificity, it represents delay in decision making, additional cost and avoidable additional exposure to radiation. Radiation exposure is important when considering implementing guidelines in patients with high‐risk PCa who often require long‐term imaging follow up or radiation treatment. BS exams give a total effective ionising radiation dose of 6.3 millisieverts (mSv).^14^ This is over twice the annual exposure of an average person living in the United Kingdom. Considering up to 25% of patients undergoing BS need additional imaging for equivocal findings, the additional radiation dose can represent more than several years of natural irradiation.

The results of this present study used real‐world clinical data and confirms that of previous research but also presents a cost‐effective and time efficient strategy for bone staging. The large cohort in our study gives a good representation of the true rate of BM in patients with high‐risk prostate cancer. The rate of BM in our cohort was 17.5%, which is equivalent if not higher than that of the literature for BS.^15^, ^16^, ^17^, ^18^, ^19^ The largest study of 47 224 patients by Falchook et al. used the SEER database to identify patients with a new diagnosis of PCa.^16^ They found that 2432/19 885 (12%) patients with high‐risk disease had BM. Smaller studies presented earlier showed rates of BM between 3% and 17%. Lecouvet et al. looked at a cohort of patients undergoing AS‐MRI for high‐risk PCa and found 24/44 (54%) positive for BM at diagnosis.^20^ This rate is considerably more than our study, but given such a small cohort of patients, these results are less robust. A similar study by Venkatachalapathy et al. comparing AS‐MRI and WB‐MRI reported 10/35 (28.5%) patients with high‐risk PCa harbouring BM.^21^

Our stringent criteria in the use of radiological T staging for high‐risk disease ensured that we selected the most appropriate patients for bone imaging. All patients in our cohort underwent mpMRI prior to AS‐MRI, which enabled complete staging. There is an argument that these two scans can be combined as part of a one‐stop examination in less than an hour, although this would likely be more appropriate in regions with a higher proportion of advanced and metastatic PCa at presentation.

An equivocal result on bone imaging is an important factor, which often delays overall staging, increases patient anxiety and increases overall cost. Our study reported a 1.2% rate of an equivocal result. When comparing with BS, our subanalysis revealed that this rate is significantly lower (*p* < 0.001) in comparison with BS data which ranges from 6 to 29%.^17^, ^20^, ^22^, ^23^ Our data for equivocal results compare with that of the literature for AS‐MRI in high‐risk PCa.^21^, ^23^, ^24^

Our study of patients with high‐risk PCa has confirmed the notion that a PSA > 20 ng/ml, locally advanced disease and Gleason score of ≥4 + 3 are independent predictors of BM which has previously been extensively reported.^25^, ^26^ This corroborates with studies, which have shown the importance of Gleason 7 morphology in demonstrating the difference between Gleason 3 + 4 and 4 + 3 in the context of prognosis. This is further illustrated by the recent endorsement of the Cambridge Prognostic Groups for risk stratification of prostate cancer by NICE.^3^, ^27^

Multiple studies looking at the ideal imaging modality for BM in PCa reported that the use of MRI would be either not feasible with respect to practicality, or too expensive. Since the current standard of care is that patients undergo mpMRI prior to prostate biopsy, it would mean units are required to have MRI scanning accessibility and capacity. All our patients were able to undergo a bone staging scan in less than 14 days, which is less than the national waiting target for cancers. We did not find a significant increased burden on our MRI service. Indeed, it is not acceptable to delay treatment in high‐risk patients waiting to rule out BM and it is therefore important to accept that if patients are waiting more than the recommended time, then AS‐MRI is not the answer. Additionally, it takes approximately 3 h for a complete BS, which is significantly more than our data for AS‐MRI, which took on average 50 min. AS‐MRI should also be strongly considered as a staging modality in the era of frequent Tc 99m shortage, which has been the case worldwide over the last few years. This has often led to significant increases in waiting times for scans.^28^

The present study demonstrates the clear benefit of AS‐MRI with respect to overall cost saving in a publicly funded health service. We demonstrated a minimum cost saving of £8406.89 over a 5 year period. Considering that up to 25% of patients undergoing BS would need additional downstream imaging for equivocal lesions, this could equate to a cost saving of up to £17 665.37, which could be better allocated to a different health resources.

Positron emission tomography (PET)‐CT with the ^68^Ga‐labelled prostate specific membrane antigen (PSMA) ligand 11 has emerged as a challenger in the staging of patients with high‐risk PCa with the most recent data from Hofman et al. showing promising results.^29^ It will however undoubtedly result in stage migration of patients, such that many patients with localised disease on conventional imaging will now have oligometastatic disease. Furthermore, up to 10% of prostate cancers are PSMA negative, which necessitates additional/alternate imaging.^30^ Until its role in the management of these patients is established, more conventional imaging will continue to be used in the initial staging of high‐risk PCa patients. Subanalysis of our patient cohort demonstrated similar rates of radical treatment compared with NPCA data when using AS‐MRI as a staging tool.^6^

The limitations of this study include its retrospective nature, although all information was accessible through an online platform and quality control was performed by two of the authors (OE, HE). There is currently no consensus for a scoring system to report AS‐MRI and therefore we recommend this in order to have better standardisation of reporting. We used AS‐MRI as opposed to WB‐MRI which deliberately limits imaging to the axial skeleton. Previous studies has shown that this approach does not result in any significant loss of accuracy in staging patients with PCa.^31^ Our preliminary cost analysis only provides an indication of the economic impact. More robust economic evaluation is warranted to assess downstream costs on patient treatment. Although our study was not designed to be comparative, our results would no doubt be strengthened if our patients also underwent BS. This study nevertheless has allowed evaluation of our current practice and provides a cost‐effective, feasible and accurate imaging modality for patients with high‐risk PCa. We therefore suggest that this is an alternative staging modality which should not be forgotten.

## CONFLICT OF INTEREST

All authors have declared no conflict of interest.

## AUTHOR CONTRIBUTIONS

Omar El‐Taji: Conceptualization; methodology; analysis; writing – original and editing. Hannah Evans: Data curation. Vandan Arora: Methodology; resources; review; editing. Suzanne Amin: Resources; review. Manal Kumar: Conceptualization; resources; review. Thiagarajan Nambi Rajan: Conceptualization; resources; supervision; validation; review and editing.
